# Sacubitril/Valsartan in Heart Failure Hospitalization: Two Pills a Day to Keep Hospitalizations Away?

**DOI:** 10.7759/cureus.37335

**Published:** 2023-04-09

**Authors:** Navya Sakhamuri, Sreekartthik Athiyaman, Bhawna Randhi, Sai Dheeraj Gutlapalli, Jingxiong Pu, Maheen F Zaidi, Maithily Patel, Lakshmi Malvika Atluri, Natalie A Gonzalez, Michael Alfonso

**Affiliations:** 1 Internal Medicine, California Institute of Behavioral Neurosciences & Psychology, Fairfield, USA; 2 Medicine, NRI Medical College, Chinakakani, IND; 3 Medicine, California Institute of Behavioral Neurosciences & Psychology, Fairfield, USA; 4 Psychiatry and Behavioral Sciences, California Institute of Behavioral Neurosciences & Psychology, Fairfield, USA; 5 Research, California Institute of Behavioral Neurosciences & Psychology, Fairfield, USA; 6 Family Medicine, California Institute of Behavioral Neurosciences & Psychology, Fairfield, USA; 7 General Surgery, California Institute of Behavioral Neurosciences & Psychology, Fairfield, USA; 8 Surgery, Dr. Pinnamaneni Siddhartha Institute of Medical Science, Gannavaram, IND; 9 Pediatrics, California Institute of Behavioral Neurosciences & Psychology, Fairfield, USA

**Keywords:** hospital re-admission, re-hospitalization, hospitalization for heart failure, heart failure with reduced ejection fraction, sacubitril-valsartan

## Abstract

Heart failure (HF) is a clinical syndrome with signs and symptoms that result from any structural or functional deterioration of ventricular filling or ejection of blood. It is the final stage of various cardiovascular diseases (e.g., coronary artery disease, hypertension, previous myocardial infarction) and remains one of the leading causes of hospitalization. It poses severe health and economic burden worldwide. Patients usually present with shortness of breath due to impaired cardiac ventricular filling and decreased cardiac output. Cardiac remodeling due to the renin-angiotensin-aldosterone system overactivation is the final pathological mechanism leading to these changes. The natriuretic peptide system is also activated to stop the remodeling. Sacubitril/valsartan, an angiotensin-receptor neprilysin inhibitor, has prompted a substantial conceptual change in HF treatment. Its primary mechanism is the inhibition of cardiac remodeling and the prevention of natriuretic peptide degradation by inhibiting the enzyme neprilysin. It is an efficacious, safe, and cost-effective therapy that improves the quality of life and survival rate in patients with HF with reduced ejection fraction (HFrEF) and HF with preserved ejection fraction. It has been demonstrated to significantly reduce hospitalization rates and rehospitalization for HF when compared to enalapril.

In this review, we have discussed the benefits of sacubitril/valsartan in treating patients with HFrEF, particularly in reducing hospitalizations and readmissions. We have also compiled studies to examine the drug’s effect on adverse cardiac events. Finally, the cost benefits of the drug and optimal dosing strategies are also reviewed. Our review article, combined with the recommendations of the 2022 American Heart Association guidelines for heart failure, strongly suggests that sacubitril/valsartan is a cost-effective strategy that reduces hospitalizations for HFrEF patients when started early with optimal doses. There is still much uncertainty regarding the optimal usage of this drug, its use in HFrEF, and the cost benefits when used alone compared with enalapril.

## Introduction and background

Heart failure (HF), a complex clinical syndrome, is a leading and increasing cause of hospitalization and mortality rate worldwide [1]. In a systematic review and meta-analysis of randomized clinical trials, Lin et al. described it as the “last battlefield” of cardiovascular diseases. The population’s prevalence rate of HF is 0.9% globally, and the prevalence rate increases significantly according to the epidemiological analysis [2]. The rate of re-admissions for HF patients also remains very high. According to an expert consensus position paper, the percentage of HF patients with readmission for all causes, either cardiac or non-cardiac, within 365 days post-discharge is as high as 44% [3]. The primary basic mechanism underlying the progression of HF is cardiac remodeling. The constant activation of the autonomic nervous system (ANS) and various cytokines such as tumor necrosis factor-alfa (TNF-α), becomes a vicious circle and deteriorates heart function [4]. The worsening of HF with reduced ejection fraction is usually associated with changes in cardiac function and structure, leading to elevated internal cardiac pressures and reduced cardiac output at rest or during stress periods [1,5]. These patients present with fatigue, breathlessness, ankle swelling, peripheral edema, elevated jugular venous pressure, or pulmonary crackles [6]. For many years, guideline-directed medical therapy (GDMT) has been routine in treating patients with heart failure with reduced ejection fraction (HFrEF) [7]. The new Golden Triangle scheme developed recently includes sacubitril/valsartan as an excellent option for treating chronic HF by replacing the renin-angiotensin system inhibitor [5]. Sacubitril/valsartan, an angiotensin receptor neprilysin inhibitor (ARNI), combines inhibition of the renin-angiotensin system and preservation of the counter-regulatory natriuretic peptide (NP) system [8]. This drug was evaluated in a double-blind, randomized, multinational trial, the Prospective Comparison of Angiotensin Receptor Neprilysin Inhibitors with ACE Inhibitors to Determine Impact on Global Mortality and Morbidity in Heart Failure trial (PARADIGM-HF trial). The trial compared sacubitril/valsartan with enalapril in patients with New York Heart Association class II-IV HF with left ventricular ejection fraction ≤40%. The drug significantly reduced cardiovascular death or HF hospitalization compared with enalapril among patients with chronic HFrEF [9-11]. It has also been proven to reduce adverse reactions such as symptomatic hypotension, worsening renal function, or angioedema in patients with HFrEF, further reducing hospitalizations [12].

After discovering that sacubitril/valsartan is the most promising drug to emerge in the field of HF treatment in the past 20 years, significant guidelines recommended switching to sacubitril/valsartan in eligible patients [9]. In July 2015, sacubitril/valsartan was approved by the United States Food and Drug Administration (US FDA) and in Europe by the European Medicines Agency (EMA) in November 2015 for use in chronic symptomatic HFrEF [10]. It is considered one of the foundational therapies, which should, where possible, be prescribed to all patients with HFrEF to minimize their risk of worsening HF and reduce hospital admissions [7]. Although the recommendations supporting the use of GDMT for HFrEF are solid, one study pointed out that registry data still show that rates of comprehensive pharmacotherapy for HFrEF care are low in usual care [9]. The prognosis in these patients is still poor, with high mortality [13].

Many eligible patients remain untreated, limiting the benefits of optimized therapy for patients with HFrEF [14]. Although there are numerous reasons for this such as symptomatic hypotension and renal failure, the biggest dilemma in standard treatment is starting the drug when there is an indication [13]. Another significant issue is that there are other contributing factors such as the presence of comorbidities, changes in the mechanisms involved in the pathogenesis of HF, the prevalence of polypharmacy/multiple prescriptions in HF patients, and modified drug properties and drug interactions in older adults [5,6]. The indications and contraindications of the drug are clear, but each patient has its singularity. The major adverse event that would cause patient non-compliance would be symptomatic hypotension; other factors such as serum potassium and worsening renal failure are of concern to the physician. If not approached correctly, there is the chance of low adherence to pharmacological treatment as a significant consequence, with an altered prognosis for HF patients.

Moreover, it should be noted that the number of people older than 80 will triple by 2050. It is important to decrease the prevalence of heart failure and its incidence to decrease morbidity, hospitalizations, and healthcare costs [6]. The medical community should make a crucial effort to optimize pharmacological regimens [15]. Sacubitril/valsartan also reduces the clinical and economic burden on these patients and can remarkably improve their quality of life [5,16]. Thus, selecting a tailored therapeutic approach according to each patient’s specific phenotypes would be beneficial [15]. Many studies have shown that the initiation of oral sacubitril/valsartan during hospitalization for HF is associated with numerous clinical outcome benefits.

In this review article, we focused on different papers to address the benefits of adding sacubitril/valsartan in reducing hospitalizations, hospital readmissions, and adverse effects, along with comparing the cost-benefit of the drug and prescribing optimal doses. In the coming years, we must see sacubitril/valsartan being brought into large-scale implementation in practice, with possible expansion of its therapeutic indications.

## Review

Mechanism of cardiac remodeling

**Figure 1:**
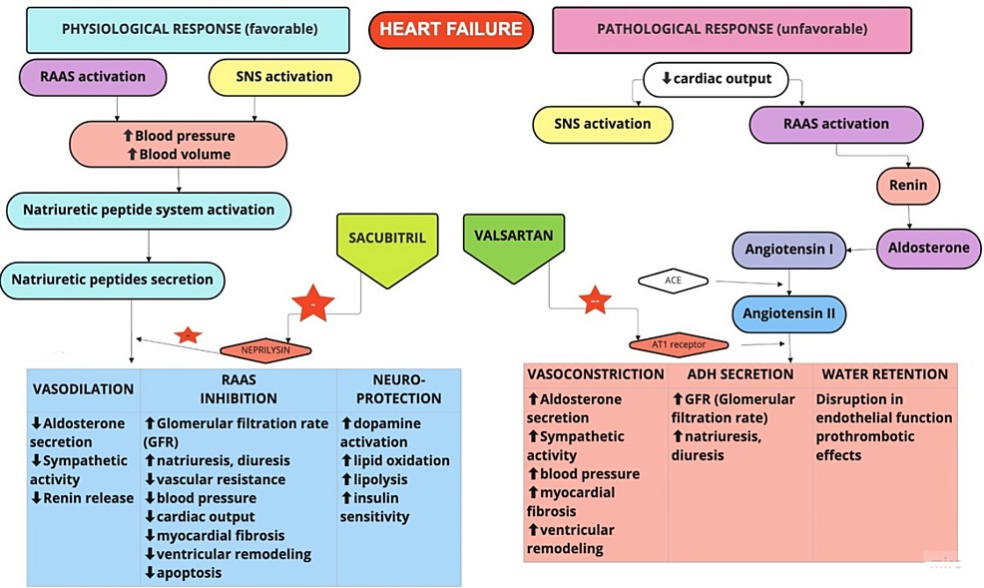
The neurohormonal mechanism and action of sacubitril/valsartan. RAAS: renin-angiotensin-aldosterone system; SNS: sympathetic nervous system; ACE: angiotensin-converting enzyme; AT1: angiotensin 1 Image credit: Navya Sakhamuri. Software used: Miro.

HF is the final result of a vicious cycle of cardiac remodeling triggered and promoted by various neuroendocrine humoral factors [4]. It begins with acute or chronic loss of cardiac function (systolic or diastolic). There is a significant loss of balance between sympathetic and parasympathetic afferent systems. Blunting of baroreflex occurs in association with hyperactive chemo and ergo reflexes. This mechanism (explained in Figure 1 below) initially aims at restoring circulatory homeostasis, but, eventually, the final result of this imbalance is an overactivation of the sympathetic nervous system (SNS) [8]. This process has adverse effects in the long term. Myocyte biology alters with the induction of cardiomyocyte apoptosis and necrosis, finally leading to myocardial fibrosis. The end effects are further changes in the left ventricular chamber geometry and left ventricular remodeling [5]. Providing optimal blood flow to the organs is the pivotal function of the heart. In these patients, the heart cannot pump enough blood to meet the body’s requirements, and thus, there is a disruption in metabolic and functional processes [6].

Based on these pathological mechanisms, drugs such as angiotensin-converting enzyme inhibitors (ACEIs), beta-blockers, mineralocorticoid receptor antagonists (MRAs), and angiotensin II receptor blockers (ARBs) have been investigated in HFrEF and were found to be beneficial. The Golden Triangle therapy for HFrEF that has been in use for years includes the renin-angiotensin system inhibitor, aldosterone receptor antagonist, and β-receptor agonist. However, some patients observed the drug effects only after taking the drugs for a long time, and the efficacy of this therapy varied from person to person [5]. Recently, this therapeutic approach has been further enhanced. Because NPs are degraded by the enzyme neprilysin, the endothelial endopeptidase, it was proposed that its inhibition could be an important therapeutic target in HF [17]. In the past years, we had four pillars in HF pharmacological treatment, which include sacubitril/valsartan as a primary option for treating chronic HF by replacing renin-angiotensin system inhibitors [5].

Mechanism of action of sacubitril/valsartan-neprilysin and angiotensin receptor inhibition

The drug sacubitril/valsartan is a sodium complex that inhibits enkephalin enzymes and angiotensin receptors, thus called the dual inhibitor. It helps dilate blood vessels and promote urinary sodium excretion, lowering blood pressure and alleviating heart failure symptoms [4]. This new pharmacological class of ARNI has prompted a substantial conceptual change in HF treatment [18]. Its primary mechanism is inhibiting cardiac remodeling and preventing NP degradation. The following sequence of events occurs: in response to volume overload and cardiac dysfunction, the failing heart tries to counteract the renin-angiotensin-aldosterone system (RAAS) and the sympathetic nervous system activation (SNA) by the natriuretic peptide system (NPS), inducing natriuresis and diuresis, exerting an antifibrotic effect at the cardiac level. This causes RAAS vasodilation and inhibition. The serum levels of NPS, atrial natriuretic peptide (ANP), and brain natriuretic peptide (BNP) increase with the worsening of HF, counterbalancing the adverse effects of RAAS and SNA overactivation. However, their efficacy is progressively reduced due to decreased availability of biologically active NPs or altered target organ responsiveness. Decreased bioavailability seems to be related to neprilysin overactivity, which catalyzes the degradation of NPs. Thus, its inhibition could lead to an increase in NP activity [2].

Sacubitril, a prodrug, and its active metabolite inhibit neprilysin, thus allowing NPs to persist longer and promote vasodilation, diuresis, and natriuresis, as well as prevent cardiac hypertrophy. At a molecular level, by augmenting the active NPs, neprilysin inhibition increases the generation of myocardial cyclic guanosine 3′5′ monophosphate, which improves myocardial relaxation and reduces hypertrophy [2]. However, neprilysin also causes the degradation of other substrates favoring remodeling, such as angiotensin II (ATII) [19]. It results in reflex activation of the RAAS system [17]. Thus, the neprilysin inhibitor sacubitril has been combined with an ATII antagonist, valsartan, to enhance its favorable effects. Valsartan acts on the RAAS, preventing vasoconstriction and decreasing aldosterone secretion and renal reabsorption of sodium, minimizing the risk of angioedema [19,20]. The changes in the cardiac remodeling indicators, six-minute walking distance, Kansas City Cardiomyopathy Questionnaire (KCCQ) scoring, and N-terminal-pro hormone BNP levels in the same patient before and after treatment were observed, which revealed improvement in prognosis and quality of life in HF patients treated with sacubitril/valsartan [4]. Multiple studies have shown that using sacubitril‐valsartan can significantly decrease the N‐terminal pro‐B‐type natriuretic peptide (NT‐proBNP). This can further enhance left atrial function [21]. Sacubitril/valsartan is among the foundational therapies alongside a beta-blocker, MRA, and a sodium-glucose cotransporter-2 (SGLT2) inhibitor. It should, whenever possible, be prescribed to all patients with HFrEF to minimize their risk of hospital admission and all-cause mortality [6,7]. The neurohormonal mechanism and action of sacubitril/valsartan are depicted in Figure 1.

Reduction in hospitalization and mortality risk

HF remains one of the dominant causes of hospitalization. There has been a steady decline in chronic HF mortality rates from 100 deaths per 100,000 population to 50 deaths per 100,000 population over the past 30 years. These numbers are from the retrospective cohorts and registries. However, it should be recognized that there is a collateral increase in HF-related hospitalizations. Close to 44% of HF patients are readmitted for cardiac and non-cardiac-related conditions within one-year post-discharge, and the duration of readmission ranges between four and six days. The first 12 weeks after discharge is considered the at-risk phase. Almost 50% of rehospitalizations arise in this phase [3]. In ambulatory patients with HFrEF who remain symptomatic even after optimal medical treatment, the previous recommendation was ACEI or ARB which is currently replaced by sacubitril/valsartan [22].

In 2014, one of the most pivotal HF trials, the Prospective Comparison of ARNI with ACEI to Determine Impact on Global Mortality and Morbidity in Heart Failure (PARADIGM-HF) trial compared sacubitril‐valsartan with enalapril in patients with HFrEF. A dose of 0.2 g ARNI LCZ696 (sacubitril) was given orally twice daily to one group and 0.01 g enalapril orally twice daily to another. After a follow-up of 27 months, the trial was stopped prematurely because of the overwhelming benefit of ARNI over enalapril. The cardiovascular mortality or HF hospitalization was 21.8% in the ARNI group vs. 26.5% in the enalapril group. The all-cause mortality was 17.0% in the ARNI group vs. 19.8% in the enalapril group. This later led to its FDA approval after a year. A European study noted that there were significant differences between the PARADIGM-HF population and European HF patients using sacubitril-valsartan after reviewing 21 articles. There were differences in the age, ischemic risk factors, target doses, and usage of MRA compared to the participants in PARADIGM-HF. A few studies compared hospitalization and mortality rate in patients receiving sacubitril-valsartan or ACEI/ARB. There was a low-to-moderate risk of bias in this comparison [23]. Sacubitril/Valsartan decreased all-cause mortality by 16% and HF hospitalization by 21% compared with standard ACEI therapy in patients with symptomatic HFrEF in another study [10,11]. Sacubitril/Valsartan also significantly improved echocardiography findings, vital signs, and biomarkers of patients with chronic HF and reduced the incidence of hyperkalemia, renal dysfunction, and other adverse reactions compared with traditional drugs to an overall reduction in hospitalizations [12].

A real-world, population-based study on Chinese patients concluded that Asian patients with HF benefitted from sacubitril/valsartan. Rates of HF-related hospitalization were low with a hazard ratio (HR) of 0.59 and a 95% confidence interval (CI) of 0.4-0.7 when compared with enalapril users, including older patients over 65 years of age [24]. A comparative study with 7,893 HFrEF patients focused on the fact that sacubitril-valsartan reduced risks of death and hospitalization for HF (HR = 0.8) compared to enalapril in ambulatory patients with HFrEF in the PARADIGM-HF trial. They concluded that in a heterogeneous cohort of patients with systolic HF (the mean follow-up time was 6.3 months), sacubitril-valsartan was associated with a reduced risk of death and hospitalization compared with ACE/ARB. Unlike other racial and ethnic groups, their outcomes with both drugs were similar in Black patients. They emphasized the fact that more research is needed to determine if there are racial differences in treatment response to sacubitril-valsartan [10]. The PIONEER-HF (Comparison of Sacubitril-Valsartan versus Enalapril on Effect on NT-proBNP in Patients Stabilized from an Acute Heart Failure Episode) clinical trial, an RCT, focused on evaluating the safety and efficacy of initiation of sacubitril-valsartan therapy among patients hospitalized for acute decompensated HF. After a follow-up of eight weeks, this multicenter, randomized, double-blind, active-controlled trial concluded that sacubitril/valsartan was more effective than enalapril in lowering the risk of HF rehospitalization or cardiovascular death (9.2% vs. 15.2%) in enrolled patients. They also concluded that sacubitril-valsartan therapy initiation led to a greater reduction in the NTproBNP concentration than enalapril therapy [25]. One meta-analysis conducted in 2021 included five relevant RCTs. The study concluded that the left ventricular ejection fraction (LVEF) improved after sacubitril-valsartan in patients with HF, with a standard mean deviation (95% CI = 1.1 [1.01, 1.19] and p < 0.00001; fixed-effects model). The left ventricular volume index (LAVI), the cardiovascular death (RR = 0.89, 95% CI = 0.83, 0.96, p = 0.003], and the rehospitalization rate of HF (RR = 0.83, 95% CI = 0.78, 0.88, p < 0.01) decreased more significantly compared to the control group [2]. The ability of sacubitril/valsartan to reduce hospitalizations is depicted by studies summarized in Table 1.

**Table 1:** The ability of sacubitril/valsartan to reduce hospitalizations is depicted by summarized studies. PARADIGM-HF: Prospective Comparison of ARNI with ACEI to Determine Impact on Global Mortality and Morbidity in Heart Failure; ACE: angiotensin-converting enzyme; ARB: angiotensin receptor blocker; ACEI: angiotensin-converting enzyme inhibitor; HF: heart failure; MI: myocardial infarction; NT-pro-BNP: N-terminal-pro hormone B-type natriuretic peptide; RCT: randomized control trial

Author	Year of publication	Purpose of study	Intervention studied	Results
Giovinazzo et al. [23]	2021	To see the real-world evidence in the European population about sacubitril/valsartan comparing it to the PARADIGM-HF trial	Sacubitril/valsartan	Significant differences noted between European patients prescribed sacubitril-valsartan and the PARADIGM-HF population, including the frequency of target dose achievement (possibly the reason for consistently high hospitalization rates even after sacubitril/valsartan use)
Tan et al. [10]	2020	To compare sacubitril/valsartan vs. ACEI/ARB in systolic heart failure	Sacubitril/Valsartan vs. ACEI	Sacubitril/Valsartan had lower risks of death and hospitalization compared with ACE/ARB in a cohort of patients with systolic HF
Zhao et al. [12]	2021	To analyze the safety and effectiveness between the early initiation of sacubitril/valsartan vs. ACEIs in patients after acute MI	Sacubitril/Valsartan vs. ACEI in MI patients	Sacubitril/Valsartan early initiation in patients after acute MI showed superiority to ACEI in reducing the risks of major adverse cardiac events but there was no difference in HF hospitalization
Pathadka et al. [24]	2021	To assess the effect of sacubitril/valsartan on survival and hospitalization risk focusing on older patients with HF	Sacubitril/Valsartan vs. ACEI in older patients	In older Asian patients with HF, in real-world settings, sacubitril/valsartan was associated with improved survival and reduced HF-related hospitalization in comparison to enalapril
Velazquez et al. [25]	2019	To see whether the initiation of sacubitril/valsartan therapy is safer and efficacious in patients who were hospitalized for acute decompensated HF	Sacubitril/Valsartan	Initiation of sacubitril/valsartan therapy had a significant reduction in the NT-proBNP concentration than enalapril therapy (which can further reduce hospitalizations and mortality)
Lin et al. [2]	2021	To assess the efficacy and safety of sacubitril-valsartan in patients with HF, relevant RCTs were analyzed	Sacubitril/Valsartan	Sacubitril/Valsartan may improve cardiac function in HF. Because a limited number of included studies are included, the study concluded that additional large sample-size RCTs are required to determine the long-term effect of cardiac function of sacubitril/valsartan in patients with HF

Reduction in adverse cardiovascular events and readmissions

HF usually occurs associated with comorbidities such as atrial fibrillation, myocardial infarction or stroke, diabetes, chronic lung disease, depression, dementia, thyroid disease, and chronic kidney/liver failure. All of these associations require additional treatment strategies [6]. Recent studies have shown that sacubitril/valsartan has shown a reduction in adverse cardiovascular events such as arrhythmias/atrial fibrillation in HFrEF patients, adverse cardiovascular events after myocardial infarction, hyperkalemia, renal dysfunction, and other adverse reactions compared with traditional drugs, all leading to an overall reduction in hospitalizations and readmissions [12,26].

In recent years, some studies have evaluated adverse events and rehospitalization. One of the adverse cardiovascular events in patients with HF is atrial fibrillation, which often coexists [26]. According to some researchers, sacubitril/valsartan has an antiarrhythmic effect by affecting three pathways of B-type natriuretic peptide, angiotensin II, and bradykinin. This review concludes that sacubitril/valsartan reduces the number of implantable cardioverter-defibrillator shocks and ventricular arrhythmias in HFrEF patients [27]. Considering that HF and atrial fibrillation have common risk factors and pathophysiologic mechanisms, another study was performed to evaluate the effects of sacubitril/valsartan on the occurrence of atrial fibrillation in patients with HF. They included six trials with a total of 15,512 patients (7,750 randomized to sacubitril/valsartan and 7,762 to control). These trials were randomized, double-blind, and active-control. Contrary to the study conducted by Wei et al., the study concluded that there was no significant difference in the prevention of atrial fibrillation between the sacubitril/valsartan group and the control group (enalapril or valsartan) in patients with HF (RR = 1.07, 95% CI = 0.95 to 1.19; I^2^ = 4%) [26].

According to a meta-analysis that compared ACEI/ARB therapy to sacubitril/valsartan therapy, sacubitril/valsartan can reduce the risks of most arrhythmias. The study reported remarkable effects in patients with high-risk factors for ventricular tachycardia (VT), ventricular fibrillation (VF), cardiac arrest, etc. [28]. A meta-analysis focused on the fact that no consensus has been reached in the research on the use of sacubitril/valsartan to treat cardiac failure after acute myocardial infarction even though it is well established that sacubitril/valsartan plays a positive role in the clinical treatment of chronic cardiac failure. This meta-analysis included five studies comprising 7,035 patients. It concluded that there is a statistically significant difference between the rehospitalization rate of the sacubitril/valsartan group and the control group. Sacubitril/valsartan showed inhibition of ventricular remodeling after acute myocardial infarction (AMI), improved cardiac function, reduced incidence of adverse cardiovascular events after myocardial infarction, rehospitalization, and mortality rates [29]. Another meta-analysis conducted by Xiong et al. had similar conclusions. Their article suggested that early administration of sacubitril-valsartan may be superior to conventional ACEI/ARB to lower the risk of hospitalization for HF, improve cardiac function, and reverse LV remodeling in patients following acute myocardial infarction [30].

Patients with ST-segment elevated MI (STEMI) are at a high risk of adverse ventricular remodeling in the initial days of follow‐up. This can lead to HF in the future. The ability of sacubitril‐valsartan to prevent abnormal cardiac fibrosis and adverse LV remodeling has been proven beneficial in HF, hypertension, and MI in animal models. However, it is still unclear whether it is effective in preventing adverse remodeling or ameliorating reverse remodeling in STEMI patients. An RCT, the PERI‐STEMI trial, will be the first CMR study to test the effects of ARNI in patients with STEMI. The results of this RCT will provide evidence on the implementation of ARNI therapy in STEMI and explain the cardiovascular protection mechanism of ARNI. The study is expected to complete in June 2027 [20].

Further research is necessary to understand the beneficial effects of sacubitril/valsartan in reducing adverse events and thus the readmission rates. A multidisciplinary approach to treatment strategies is necessary for HF patients to reduce exacerbations, hospital readmission rates, and mortality. The treatment strategy must be personalized for each HF patient and reviewed by the healthcare team periodically tracking symptoms. Additional focus is needed on patient education regarding a low salt diet, regular exercise as tolerated, and smoking and alcohol cessation. It is also crucial for healthcare providers and patients to be on the lookout for alarming signs and symptoms of decompensation such as dyspnea, fatigue, ankle swelling, and sudden weight changes [6]. The efficacy of sacubitril/valsartan in reducing adverse events and readmissions is demonstrated by studies summarized in Table 2.

**Table 2:** Summary of studies conducted on sacubitril/valsartan in reducing adverse events and readmissions. HF: heart failure; HFrEF: heart failure with reduced ejection fraction; RCT: randomized controlled trial; ACE: angiotensin-converting enzyme; ARB: angiotensin receptor blocker; VF: ventricular fibrillation; VT: ventricular tachycardia; LV: left ventricle; AMI: acute myocardial infarction; STEMI: ST-segment elevation myocardial infarction

Author	Year of publication	Purpose of study	Adverse events of HF studied	Results
Liu et al. [26]	2022	To assess the use of sacubitril/valsartan on atrial fibrillation occurrence in patients with HF	Atrial fibrillation	No difference was seen between Sacubitril/valsartan and enalapril or valsartan in preventing the occurrence of atrial fibrillation in patients with HF
Wei et al. [27]	2022	To see if sacubitril/valsartan use can reduce ventricular arrhythmias	Ventricular arrhythmia	Sacubitril/valsartan reduces the number of ventricular arrhythmias and implantable cardioverter-defibrillator shocks in HFrEF patients, thus reducing rehospitalization and emergency treatment rate
Wang et al. [28]	2022	Meta-analysis of RCTs to know the preventative effect of sacubitril/valsartan on lowering the risk of arrhythmias	Arrhythmias	Sacubitril/Valsartan can reduce the risks of most arrhythmias when compared to ACEI/ARBs. Remarkable effects were seen in patients with high-risk factors for VF, VT, and cardiac arrest in patients with HFrEF
Zhang et al. [29]	2022	To see if the early introduction of sacubitril/valsartan after AMI has any clinical efficacy and safety	AMI	Sacubitril/Valsartan inhibits ventricular remodeling, thus reducing the incidence of adverse cardiovascular events, the rehospitalization rate, and the mortality rate after myocardial infarction
Xiong et al. [30]	2021	To explore whether sacubitril-valsartan can potentially improve the prognosis, cardiac function, and LV remodeling in patients after AMI	AMI	Early introduction of sacubitril-valsartan may have an upper hand over conventional ACEI/ARB to lower the hospitalization risk for HF, reverse the LV remodeling improve overall cardiac function in patients following AMI
Diao et al. [20]	2021	PERI-STEMI trial tries to interpret whether sacubitril-valsartan is more effective in the prevention of adverse LV remodeling for patients with STEMI than enalapril	STEMI	The expected completion date is June 2027

Sacubitril/Valsartan in heart failure with reduced ejection fraction versus heart failure with preserved ejection fraction

Multiple pharmacological and non-pharmacological therapies of HFrEF have proven beneficial, and it has been established that sacubitril/valsartan has an overall prognostic benefit compared to others. On the other hand, the outcome of patients with HFpEF remains poor [8]. Moreover, diagnosing and treating HFpEF and managing advanced and acute HF remains challenging. Although the progress remains slow concerning HFpEF, sacubitril/valsartan holds great promise for this condition and SGLT2 inhibitors, and there are currently large clinical trials underway (Prospective Comparison of ARNI vs Comorbidity-Associated Conventional Therapy on Quality of Life and Exercise Capacity (PARALLAX trial)). The recent progress in diagnostic methods for accurate HFpEF diagnosis will most likely assist us in future clinical trials. This can potentially lead us to find effective therapies [18]. A systematic review of double-blind, RCTs compared sacubitril/valsartan to a reference drug in HFrEF and HFpEF patients. In patients with HFrEF (two RCTs, 4,627 patients), all-cause mortality was 16% on sacubitril/valsartan and 18% on enalapril, with an RR of 0.85 and CI of 0.78, 0.93. In patients with HFpEF (two RCTs, 5,097 patients), it was 13% vs. 14%, with no statistical difference. Thus, they concluded that sacubitril/valsartan reduces all-cause mortality in HFrEF patients, but further data are needed in patients with HFpEF [19].

On the other hand, another RCT focused on HF with preserved ejection fraction and conducted a post hoc analysis of the prospective comparison of ARNI with ARB Global Outcomes in the HFpEF trial (PARAGON-HF). They assessed the patients with HFpEF (≥45%) and concluded that compared with valsartan, absolute risk reduction (ARR) values with sacubitril/valsartan were more prominent in patients enrolled early after hospitalization. ARR was 6.4% in patients enrolled under 30 days, 4.6% in patients enrolled in 31 to 90 days, and 3.4% in patients enrolled in 91 to 180 days, whereas no risk reduction was observed in patients screened >180 days or who were never hospitalized [31]. Because drugs preventing cardiac remodeling are key to halting the progression of ventricular functional impairment and, in turn, improving the prognosis of patients with HFrEF, it is crucial to initiate the treatment early. The drugs that slow the progression of damaging cardiac remodeling are ACEI, ARB II, and MRA, whereas the drugs that induce reverse cardiac remodeling, causing an appreciable decrease in ventricular volumes and an increase in LVEF are beta-blockers, cardiac resynchronization therapy, and ARNI [17]. Overall, PARAGON-HF is the only study that evaluated sacubitril/valsartan in HFpEF. There was a trend seen in the study but there is no statistical significance. Further research is necessary on the use of sacubitril/valsartan in HFpEF patients [19].

Cost benefits with sacubitril/valsartan use

The paramount burden increasing costs of HF treatment is hospitalization leading to enormous costs to the healthcare system. Initiation of sacubitril/valsartan during hospitalization is beneficial in reducing this burden. It saves approximately $452 per year compared with continuing enalapril. If initiated early in the hospitalization course instead of late or post-discharge initiation, it can save $811 per year. Inpatient initiation of the drug was associated with an improved overall cost-benefit ratio of $21,532 per quality-adjusted life-year (QALY) compared with continuous enalapril treatment for a lifetime [3].

A retrospective cohort study funded by Novartis included patients with HFrEF who had never taken any RAAS inhibitor before. They compared real-world outcomes of ARNI (sacubitril/valsartan) vs. ACEI or ARB therapy in these patients. They collected the data from the Optum Electronic Health Records database in the United States Patients who had newly initiated sacubitril/valsartan or ACEI/ARB between July 2015, and March 2019 were included. Rates of all-cause, HF, and cardiovascular hospitalizations and the composite of HF hospitalization or emergency room (ER) visits were measured. The study concluded that in patients with HFrEF who have never taken any RAAS inhibitor, sacubitril/valsartan initiation reduced the clinical burden by decreasing total hospitalizations and ER visits [16].

A study in Thailand compared the cost-benefit of sacubitril-valsartan with enalapril in HF patients with acute decompensation. The findings of the study suggested that treatment of acute HF with sacubitril-valsartan combination therapy compared with enalapril had an incremental cost-effectiveness ratio (ICER) value of US$ 3451.26 per QALY. It showed more QALYs (4.969 for sacubitril/valsartan vs. 4.755 for enalapril). This is considered a cost-effective approach in Thailand. Early start of sacubitril-valsartan treatment would be more cost-effective than delayed treatment, with ICERs of US$3451.26 per QALY and US$4461.71 per QALY, respectively. Sacubitril-valsartan was shown to be cost-effective in patients with acute HF. The results, however, are highly dependent on long-term cardiovascular mortality, and they apply only to a few countries such as Thailand or countries with similarly structured healthcare systems. It has been concluded that, even though model-based analyses can help understand cost and effectiveness drivers, long-term registries should be used to decrease the ambiguity around long-term mortality [32].

Prescribing optimal doses to reduce readmissions

By now, it has been well established that initiation of oral GDMT during hospitalization for HF is associated with numerous clinical benefits. In the same way, the continuation of oral GDMT during hospitalization for HF lowered the risk of post-discharge death and readmission compared with discontinuation in many registries. The American Heart Association (AHA) made a report on Guidelines for the Management of Heart Failure. They evaluated the data from different sources and found that it cannot be assumed that oral GDMT will be initiated or optimized after hospitalization for HFrEF. Here are some examples. According to the data from the CHAMP-HF (Change the Management of Patients with Heart Failure) registry, among the eligible patients with HFrEF, only 73%, 66%, and 33% were prescribed ACEi-ARB-ARNi, beta-blockers, and MRA therapy, respectively. According to claims data, within 30 days post-index hospitalization, roughly 42% of patients were not prescribed any GDMT. Within one year post-hospitalization, 45% are prescribed either no oral GDMT or monotherapy. In managing patients with HFrEF in the community, they concluded that very few receive target doses of oral GDMT. Furthermore, most patients with HFrEF have no changes to oral GDMT over 12 months, even after being discharged on suboptimal doses or without GDMT [33].

An observational study used data from three hospitals in China to analyze real-world treatment patterns of sacubitril/valsartan. This non-interventional retrospective cohort study was conducted on 267 patients with HF who were prescribed sacubitril/valsartan from three tertiary hospitals in China between January 2018 and June 2020. They evaluated dose titration patterns of sacubitril/valsartan during these six months. They found out that at the end of six months, only 4.5% were prescribed with a target dose of 49 mg sacubitril and 51 mg valsartan (as sacubitril-valsartan sodium salt complex) bid, 27% of patients with 12/13 mg bid, 63.7% with 24/26 mg bid, and 4.8% were not prescribed according to the recommended dose. In six months, only one dose titration record has been noted in less than 10% of patients. Medication persistence was observed across the drug doses, and throughout the six months, only 15.7% of patients discontinued sacubitril/valsartan. Thus, they finally concluded that in China, most patients prescribed sacubitril/valsartan are not initiated on the optimal dose nor up-titrated or monitored according to drug instructions. However, good adherence to the drug was observed [34]. A study suggested a proportional relationship between drug concentrations and achieved benefits. Patients treated with lower doses of sacubitril/valsartan had a higher risk of cardiovascular events than those who were maintained at the maximal recommended dose of 200 mg twice daily [15]. Some studies suggested implementing new substances for HFrEF therapy. One of their examples is that administering a drug dose in one tablet form provides a faster therapeutic effect. They concluded that it helps formulate simple treatment rules in most cases [13].

A viewpoint expert consensus position paper concluded that sacubitril/valsartan implementation during hospitalization remains substandard. They evaluated the role of sacubitril/valsartan, considering different types of HF. This is mainly due to the absence of a personalized plan for switching to oral medications and the lack of particular guidelines regarding dose selection and uptitration. It has also been found that there is much uncertainty regarding patient eligibility. Intending to improve efficiencies of care and resource utilization, this review proposed clinical recommendations, along with a proper plan, to encourage and facilitate sacubitril/valsartan administration during hospitalization [3].

Although guidelines have changed worldwide to include sacubitril/valsartan for HFrEF patients, even now, years after the PARADIGM-HF trial, there remains some uncertainty regarding when to start sacubitril/valsartan and in whom. To provide patients with comprehensible information regarding one treatment’s potential benefits compared to another, even though more data are needed, we can consider the treatment’s estimated long-term effects as a helpful adjunct to clinical trial results [18].

Limitations

It has been challenging to define search criteria to find relevant journals and studies, which seems to be due to the limited use of the drug in real-world settings, even though many studies have proven its efficacy in reducing hospitalizations. Therefore, the designs and outcome measures of the studies included were heterogeneous. The samples included in the studies are relatively different and did not specify the associated comorbidities. Therefore, the findings cannot be generalized to the entire population. Although our study findings are encouraging, it is unclear whether similar results would be seen in those with less clinical stability and more advanced HF complicated by hypotension and renal dysfunction. Additional work is necessary to understand better and identify issues such as long-term safety in human and animal models.

## Conclusions

We have concluded that sacubitril/valsartan is an excellent drug for reducing hospitalizations and improving the overall quality of life in patients with HFrEF by improving cardiac structure and function. It reduces the rate of adverse cardiovascular events and thus the readmission rates when given early in the course with optimal dosage. The dose has to be correctly titrated on the follow-up. There is a proven benefit of the drug in reducing hospitalizations in the HFrEF group, but there is not enough information concerning the HFpEF group. More research is necessary on the same. By decreasing total hospitalizations and ER visits, sacubitril/valsartan also reduces the clinical and economic burden. The cost-benefit of this drug is found to be higher when initiated early compared to enalapril. More research is needed on optimal dosing, medication treatment costs alone, and the use of the drug in HFpEF patients. Although the drug has proven its potential in many studies, including extensive clinical trials, the optimal use of this drug in the clinical setting remains at stake. Clearer data must be made available to healthcare providers and patients. To reduce hospitalizations in HF patients, an interdisciplinary approach to treatment strategies is mandatory. The treatment strategy must be individualized for each HF patient and monitored and reviewed by the healthcare team. With carefully designed studies, additional work is necessary to understand better and identify issues such as long-term safety in human and animal models. The discovery of sacubitril/valsartan has been a milestone and a crucial component of disease-modifying medical therapy in managing chronic HFrEF. The future potential of this drug looks good.
